# Insulin signaling regulates neurite growth during metamorphic neuronal remodeling

**DOI:** 10.1242/bio.20136437

**Published:** 2013-12-11

**Authors:** Tingting Gu, Tao Zhao, Randall S. Hewes

**Affiliations:** Department of Biology, University of Oklahoma, Norman, OK 73019, USA

**Keywords:** Insulin, Neuronal remodeling, Metamorphosis, Organizational growth, Allometric growth, Axon branching

## Abstract

Although the growth capacity of mature neurons is often limited, some neurons can shift through largely unknown mechanisms from stable maintenance growth to dynamic, organizational growth (e.g. to repair injury, or during development transitions). During insect metamorphosis, many terminally differentiated larval neurons undergo extensive remodeling, involving elimination of larval neurites and outgrowth and elaboration of adult-specific projections. Here, we show in the fruit fly, *Drosophila melanogaster* (Meigen), that a metamorphosis-specific increase in insulin signaling promotes neuronal growth and axon branching after prolonged stability during the larval stages. FOXO, a negative effector in the insulin signaling pathway, blocked metamorphic growth of peptidergic neurons that secrete the neuropeptides CCAP and bursicon. RNA interference and CCAP/bursicon cell-targeted expression of dominant-negative constructs for other components of the insulin signaling pathway (InR, Pi3K92E, Akt1, S6K) also partially suppressed the growth of the CCAP/bursicon neuron somata and neurite arbor. In contrast, expression of wild-type or constitutively active forms of InR, Pi3K92E, Akt1, Rheb, and TOR, as well as RNA interference for negative regulators of insulin signaling (PTEN, FOXO), stimulated overgrowth. Interestingly, InR displayed little effect on larval CCAP/bursicon neuron growth, in contrast to its strong effects during metamorphosis. Manipulations of insulin signaling in many other peptidergic neurons revealed generalized growth stimulation during metamorphosis, but not during larval development. These findings reveal a fundamental shift in growth control mechanisms when mature, differentiated neurons enter a new phase of organizational growth. Moreover, they highlight strong evolutionarily conservation of insulin signaling in neuronal growth regulation.

## Introduction

Although differentiated neurons have relatively stable morphologies, they nevertheless undergo dynamic structural changes in order to sustain their functions. These maintenance growth processes include the recycling of membrane and other cellular components (Kelly, 1993; Zimmermann et al., 1993), the expansion or retraction of synaptic contacts (Zito et al., 1999; Eaton et al., 2002), and growth in proportion to changes in tissue size (Bentley and Toroian-Raymond, 1981; Loesch et al., 2010). For example, mouse lumbar spinal motorneurons, *D. melanogaster* sensory neurons, and *Manduca sexta* (tobacco hornworm) larval motorneurons all display post-embryonic growth in proportion to body size while maintaining their topologies (Truman and Reiss, 1988; Li et al., 2005; Parrish et al., 2009).

Neurons display another, organizational form of growth associated with axonal and dendritic pathfinding and elaboration of new neuronal arbors. Organizational growth is normally restricted to initial neuronal differentiation, but it also occurs in fully differentiated neurons under certain situations, including puberty, insect metamorphosis, and seasonal changes in bird song control centers, and in response to injury, stroke, or neurological disease (Levine and Truman, 1982; Finger and Almli, 1985; Brenowitz, 2004; Blakemore and Choudhury, 2006; Benowitz and Carmichael, 2010). Mature neurons vary widely in their capacities to undergo organizational growth (Holm and Isacson, 1999; Goldberg and Barres, 2000), and the factors contributing to these differences are poorly understood. Nevertheless, several known regulators of organizational growth, such as neurotrophic factors, cell adhesion molecules, and modulators of cytoskeletal reorganization, are associated with neurodegenerative diseases (Mattson, 1990; Cotman et al., 1998; Kao et al., 2010). There is intense interest in finding ways to stimulate organizational growth in mature neurons to counter nervous system damage (Maier and Schwab, 2006; Mattson, 2008; Zhang et al., 2008).

Insect neurons are a powerful model for examining transitions between maintenance and organizational growth and for studying differences in the control of these distinct growth processes. In holometabolous insects, fully differentiated larval neurons exhibit maintenance growth during the larval stages and a second, post-embryonic phase of organizational growth during metamorphosis. During this latter phase, many larval neurons are retained and undergo significant structural remodeling; larval axons and dendrites (neurites) are pruned back, and this is followed by the outgrowth of adult projections (Truman, 1990).

The *D. melanogaster* CCAP/bursicon neurons provide an excellent genetic model to examine post-embryonic organizational growth (Zhao et al., 2008). These neurons secrete multiple neuropeptides, including bursicon and crustacean cardioactive peptide (CCAP), to regulate molting behaviors (Park et al., 2003; Dewey et al., 2004). In larvae, the CCAP/bursicon neurons consist of at least 3 pairs of neurons in the brain (with one or both peptides), 3–4 pairs of neurons in the lateral subesophageal ganglia, and at least 21 pairs in the ventral nerve cord (VNC) (Hodge et al., 2005; Vömel and Wegener, 2007; Zhao et al., 2008). Most of these neurons project within the VNC, but several abdominal pairs send efferent projections via segmental nerves to the periphery to terminate on larval body wall muscles, where they form neuroendocrine endings (Hodge et al., 2005; Vömel and Wegener, 2007; Zhao et al., 2008). Additional efferents terminate in the more posterior abdominal nerves (this study). The morphology of the CCAP/bursicon neurons is maintained throughout larval development, but they grow more than two-fold in size in proportion to the overall larval growth (supplementary material Fig. S1). During metamorphosis, the larval axons and dendrites are pruned back almost to the cell bodies, followed by outgrowth of adult-specific neurites, which include a peripheral, tree-like axonal arbor with *en passant* neuroendocrine boutons (Zhao et al., 2008).

The CCAP/bursicon neurons are essential for completion of two events in the life cycle: pupal ecdysis, at the onset of metamorphosis, and wing expansion, which occurs after metamorphosis is completed and the adult has eclosed (McNabb et al., 1997; Park et al., 2003). Disruption of the CCAP/bursicon neurons prior to pupal ecdysis produces animals that fail to evert the adult head from the thorax and to fully elongate the adult legs and wings. Later perturbation of the CCAP/bursicon neurons during metamorphosis leads to viable, fertile adults with permanently unexpanded wings (Zhao et al., 2008). Although pupal ecdysis and wing expansion behaviors each last only a few minutes, the resulting morphological defects persist for days and are easy to score (Zhao et al., 2008). Therefore, we can conduct large-scale genetic screens for factors that contribute specifically to organizational growth by selecting for genetic alterations in the CCAP/bursicon neurons that preferentially disrupt wing expansion.

In vertebrates, insulin and insulin-like growth factor 1 (IGF-1) are both important regulators of nervous system growth and maturation. IGF-1 has well-established functions in controlling neuronal growth, survival, and plasticity, and in maintaining cognitive function throughout the lifespan (Aleman and Torres-Alemán, 2009). Insulin, a critical regulator of nutrient homeostasis, has been implicated more recently in the morphogenesis and function of the central nervous system (CNS) (Chiu and Cline, 2010; Huang et al., 2010). Several neuronal cell culture studies have revealed a role for insulin receptor signaling in regulating neurite growth (Govind et al., 2001; Choi et al., 2005), and *in vivo* studies in retinotectal circuits of the frog *Xenopus laevis* have shown that insulin receptor signaling is required for dendritic arborization (Chiu et al., 2008).

The insulin and insulin-like growth factor signaling (IIS) pathway has been highly conserved throughout evolution. The structure of the mature peptide hormones is shared by mollusks, nematodes, insects, and humans (Conlon, 2001; Claeys et al., 2002), and these peptides act on a small family of closely related receptor tyrosine kinases that stimulate a canonical intracellular signaling pathway (Claeys et al., 2002). In *Drosophila*, the insulin-like peptides are encoded by eight genes (*dilp1–8*) and are produced in the CNS, gut, imaginal discs, and fat body (Brogiolo et al., 2001; Colombani et al., 2012; Garelli et al., 2012). Once secreted, all of the DILPs are thought to bind and activate a single *Drosophila* insulin-like receptor (InR) (Brogiolo et al., 2001), which in turn activates insulin receptor substrate (IRS). IRS stimulates a series of kinases, including phosphatidylinositol 3-kinase (PI3K) and Akt/protein kinase B, to regulate metabolism, cell and tissue growth, longevity, and neuronal properties (Ikeya et al., 2002; Rulifson et al., 2002; Broughton and Partridge, 2009; Naganos et al., 2012).

Here, we examined the role of IIS in growth of the CCAP/bursicon neurons. Our results showed that signaling through InR strongly regulates the organizational growth of the CCAP/bursicon neuron cell bodies and neurite arbor during metamorphosis, but IIS plays only a small role in larval maintenance growth. We tested whether IIS regulates the growth of other peptidergic CNS neurons, and in most cases, the organizational growth seen during metamorphosis was substantially more sensitive to IIS than larval maintenance growth. These findings reveal a fundamental shift in growth control mechanisms as many neurons are remodeled, and they highlight an important role of IIS in this process.

## Materials and Methods

### Fly strains, genetic manipulations, and scoring

Fly stocks were cultured on a standard cornmeal–yeast–agar medium, and crosses were performed at 25°C. The following strains were obtained from the Bloomington Drosophila Stock Center: *ccap-Gal4* (y* w*;P{ccap-Gal4.P}16; FBti0037998); *UAS-InR* (*y^1^ w^1118^; P{UAS-InR.Exel}2*; FBst0008262); *UAS-InR^act^* (*y^1^ w^1118^; P{UAS-InR.R418P}2*; FBst0008250); *UAS-InR^DN^* (*y^1^ w^1118^; P{UAS-InR.K1409A}2*; FBst0008259); *UAS-PI3K* (*y^1^ w^1118^; P{UAS-Pi3K92E.Exel}2*; FBst0008286); *UAS-PI3K^act^* (*P{UAS-Pi3K92E.CAAX}1, y^1^ w^1118^*; FBst0008294); *UAS-PI3K^DN^* (*P{UAS-Pi3K92E.A2860C}1, y^1^ w^1118^*; FBst0008288); *UAS-Akt* (*P{UAS-Akt1.Exel}1, y^1^ w^1118^*; FBst0008192); *UAS-PTEN^RNAi^* (*w^1118^; P{UAS-Pten.dsRNA.Exel}3*; FBst0008550); *UAS-Rheb* (y^1^ w*; P{Mae-UAS.6.11}Rheb^LA01053^/TM3, Sb^1^ Ser^1^; FBst0022248); *UAS-S6K* (*w^1118^; P{UAS-S6k.M}2/CyO*; FBst0006910); *CyO, tubPGal80* (w*; l(2)DTS91^1^ noc^Sco^/CyO, P{tubP-Gal80}OV2; FBst0009491); and Oregon-R (wild type; FBst0004269). Other RNAi lines were obtained from the Vienna Drosophila RNAi Center. *UAS-foxo^w+[m3-1]^* and *UAS-foxo^TM[f3-9]^* were kindly provided by Marc Tatar (Providence, Rhode Island, USA). *w; bursicon-Gal4[P12]* was made by Willi Honegger (Nashville, Tennessee, USA) and provided by Ben White (Bethesda, Maryland, USA). w*, UAS-Dcr-2 was made by Stephan Thor (Linkoping, Sweden) by mobilizing a *UAS-Dcr-2* insertion (FBti0101430) (Dietzl et al., 2007) to a new X chromosome location to enhance the effect of RNAi, and that line was kindly provided by Paul Taghert (St Louis, Missouri, USA).

Wing expansion defects were scored as unexpanded wings (UEW), partially expanded wings (PEW), and expanded wings as described (Luan et al., 2006b).

### Immunostaining

Immunostaining was performed on isolated CNS or on whole-animal fillets of wandering 3^rd^ instar larvae or staged pupae (Bainbridge and Bownes, 1981) as described (Hewes et al., 2006). Primary antisera were used overnight at 4°C and were directed against the following proteins: CCAP (1:4000, PFA/PA) (Park et al., 2003), Bursicon α-subunit (1:5000, PFA/PA) (Luan et al., 2006a), DILP7 (1:1000, PFA/PA) (Yang et al., 2008), and FOXO (1:1000, PFA) (Puig et al., 2003). The FOXO antibody was used to confirm overexpression of FOXO in *ccap*>FOXO animals (data not shown). All tissues were mounted with Vectashield (Vector Labs, Burlingame, CA) for observation using an Olympus FluoView FV500 confocal microscope (Center Valley, PA).

### Staining quantification

Confocal image quantification was performed as described (Hewes et al., 2006; Zhao et al., 2008), and the images shown in the figures are representative of the mean values. The same confocal scanning settings, which were optimized to avoid image saturation, were used for all preparations within each experiment. For quantification of cell soma area, we converted the confocal Z-series stacks to maximum projection images, manually traced the cell border, and obtained a count for the bordered pixels in Adobe Photoshop. For quantification of arbor area, we used the inversion and threshold functions in Adobe Photoshop (with the same threshold of 235 for all images) to convert the background to white and all remaining pixels (arbor and somata) to black. The somata and any obvious artifacts were manually cut from each image, and then we obtained a count of the black pixels. Although we used the same settings for all images within an experiment, the intensity of images varied from experiment to experiment, necessitating the use of different scan settings. Therefore, results from different experiments are not comparable. To quantify the density of peripheral axon branches within a standardized field, branches were counted manually within a 785 pixel × 415 pixel window that was centered over the arbor with the top edge aligned with the first major branch point. To correct for differences in animal size, counts of bouton numbers at the larval CCAP/bursicon cell NMJs were normalized by the cross-sectional area measured for muscle 6 in abdominal segment 4. However, the size of muscle 6 did not vary significantly, and the results would have been the same without normalization. Statistical tests were performed using NCSS-2001 software (NCSS, Kaysville, UT) or at http://www.vassarstats.net, and a level of significance of 0.05 was used for all *post-hoc* tests performed following ANOVAs. All data are presented as means ± s.e.m. The numbers of animals for each genotype are indicated with each histogram bar in the figures. *P<0.05, **P<0.01, ***P<0.001.

## Results

### Overexpression of *foxo* disrupted metamorphic growth of the CCAP/bursicon neurons

In a previous genetic screen, we found that overexpression of the *foxo* transcription factor (*forkhead box, sub-group O*; FlyBase ID FBgn0038197) in the CCAP/bursicon neurons disrupted normal wing expansion (Zhao et al., 2008). Since FOXO is a negative regulator of IIS (Puig et al., 2003), we first examined the cellular effects of FOXO overexpression and investigated the timing and extent of IIS regulation of CCAP/bursicon neuron growth in larvae and during metamorphosis.

**Fig. 1.**
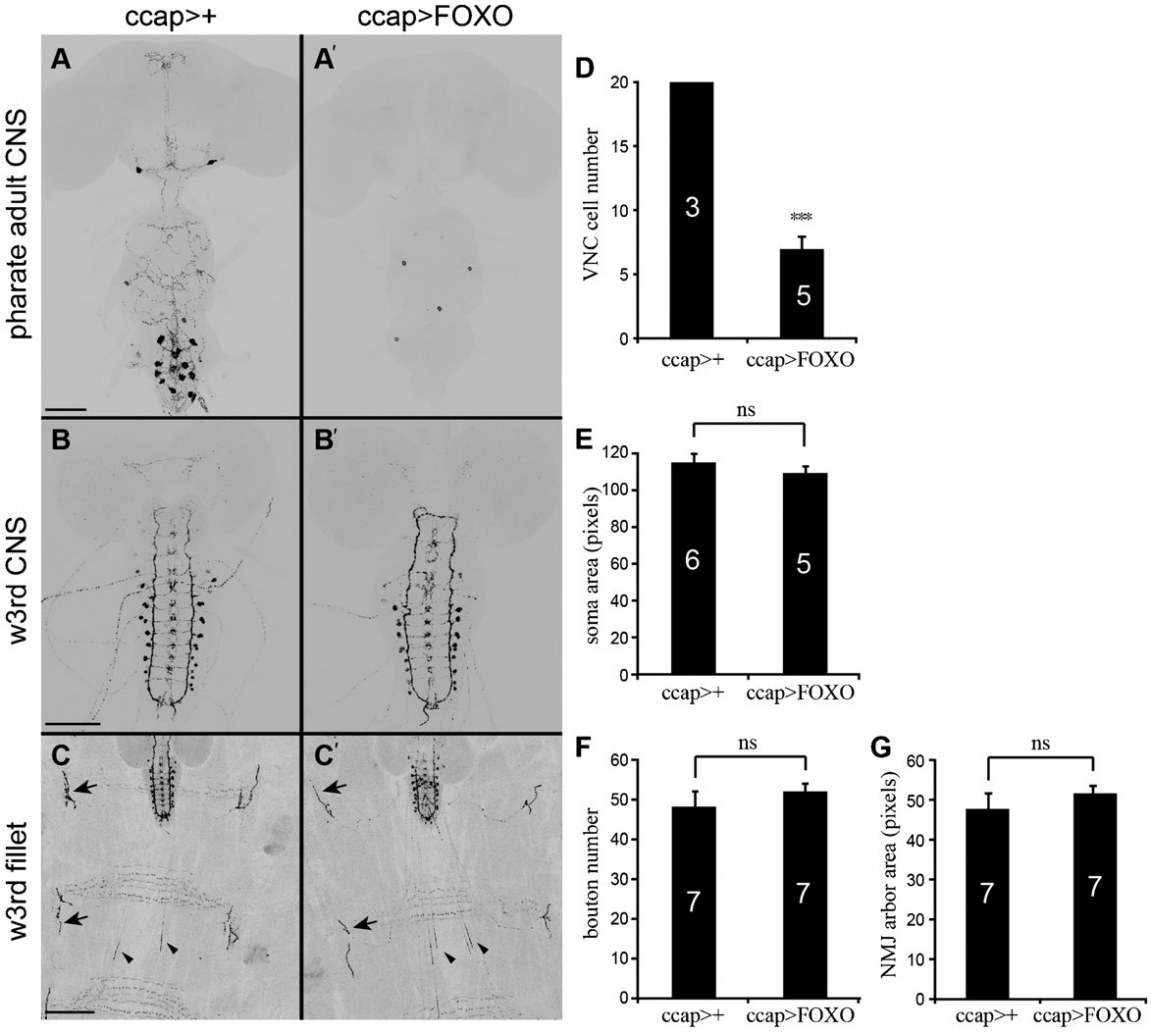
Overexpression of *foxo* disrupted pharate adult CCAP/bursicon neuron morphology. (A) Cell-targeted expression of *foxo* in the CCAP/bursicon neurons (A′) caused the loss of most adult-specific neurites and disappearance (or reduced immunostaining) of many cell bodies at the P14 pharate adult stage. Cells were labeled by anti-bursicon immunostaining, and the control genotype (*ccap*>+: *ccap-Gal4/+*) is shown in panel A. (B,C) In wandering third instar (w3^rd^) larvae, the arrangement of CCAP/bursicon somata in the CNS, and the morphology of central neurites (B′) and the peripheral axon arbor (C′), were largely normal. (D–G) Quantification (with Student's *t*-tests) of cellular properties for the experiments shown in panels A–C. Overexpression of *foxo* significantly reduced CCAP/bursicon cell number in P14 pharate adults (D) (*P*<0.0001), but there was no change in larval soma size (E) (*P*>0.05), larval NMJ bouton number (F) (*P* = 0.35), or NMJ size (G) (*P*>0.05). Arrows, selected NMJ-like endings on muscles 12 and 13; arrowheads, efferents that terminate within abdominal nerves. ns, not significant. Scale bars: 100 µm (A,B), 50 µm (C).

Following single crosses, all flies expressing *UAS-foxo* under the control of a *ccap-Gal4* driver (*ccap*>FOXO) had completely folded wings (*n* = 122). Since the CCAP/bursicon neurons are essential for initiation of wing expansion behavior (Park et al., 2003; Dewey et al., 2004), this result suggested that *foxo* overexpression disrupts the development, function, or survival of these neurons. To test this hypothesis, we performed anti-bursicon immunostaining on stage P14 pharate adult CNS (Bainbridge and Bownes, 1981) (Fig. 1). We observed a 65% reduction in the number of bursicon-immunopositive somata throughout the CNS (Fig. 1A,D), with loss of 71% of the abdominal bursicon neurons (B_AG_) (supplementary material Fig. S2), and the remaining cells displayed abnormal morphology, with reduced soma sizes and peptide expression, and a near complete loss of central and peripheral neurites (Fig. 1A′). All of the B_AG_ also express CCAP (and there are ∼14 additional abdominal neurons that express CCAP only (Luan et al., 2006b). We observed a 45% loss of CCAP neuron somata after *foxo* overexpression using membrane-associated mCD8::GFP as the cellular marker (*ccap*>FOXO, mCD8::GFP), and most of the remaining cells expressed GFP but not bursicon (supplementary material Fig. S2). Thus, *foxo* overexpression resulted in the loss of most bursicon neurons.

Less severe phenotypes were produced by *foxo* overexpression with a second driver, *bursicon-Gal4*, although the level of GFP expression was significantly higher than the expression with *ccap-Gal4* (supplementary material Fig. S3), and a later experiment involving manipulation of the insulin receptor (see below and supplementary material Fig. S4B,D) also showed no difference in the efficacy of these two drivers. In pharate adults, 78% of the CCAP/bursicon neurons with *foxo* overexpression driven by *bursicon-Gal4* remained (supplementary material Fig. S3), albeit with smaller somata and reduced branching in the peripheral axon arbor. In addition, the pharate adult somata displayed a linear arrangement (supplementary material Fig. S3A) reminiscent of the larval stages (Fig. 1B). We speculate that differences between the responses of these neurons to *foxo* overexpression driven by *ccap-Gal4* versus *bursicon-Gal4* are due either to cell–cell interactions (there are fewer cells in the *bursicon-Gal4* pattern), the timing of transgene expression mediated by these two drivers, insertion position effects, or differences in genetic backgrounds of these strains.

We then asked whether *foxo* overexpression disrupted the earlier development of the CCAP/bursicon neurons (Fig. 1B–G). We conducted anti-bursicon immunostaining on wandering 3^rd^ instar stage larvae, which had just initiated metamorphosis. All CCAP/bursicon cell somata were present. Moreover, there were no statistically significant changes in soma area (Fig. 1E), bouton number at the larval neuromuscular junctions (NMJs) (Fig. 1F), or area covered by each NMJ (Fig. 1G). Therefore, *foxo* overexpression specifically inhibited growth of the CCAP/bursicon neurons during metamorphosis.

During metamorphic remodeling of the CCAP/bursicon neurons, pruning of larval neurites peaks ∼12 hours after puparium formation (APF) and continues until ∼30 hours APF. Peak outgrowth of adult neurites occurs at 36–54 hours APF, and outgrowth is largely completed at ∼60 hours APF (Zhao et al., 2008). To determine whether pruning and/or outgrowth were disrupted by *foxo* overexpression, we examined anti-bursicon immunostaining of the CCAP/bursicon neurons at key remodeling stages. There were no changes observed at 0 hours APF, near the onset of metamorphosis. However, at 24 hours APF, when pruning of larval neurites is largely complete (Zhao et al., 2008), we observed comparable pruning in *foxo*-overexpressing cells (*ccap*>FOXO) and controls (*ccap>+*) (supplementary material Fig. S5). At 48 hours APF, when pruning in control cells is complete and adult neurite outgrowth is well underway (Zhao et al., 2008), *foxo* overexpressing cells displayed reduced soma sizes (supplementary material Fig. S5) and a much smaller and less branched peripheral axon arbor (data not shown). Therefore, *foxo* overexpression spared neurite pruning, but the growth (or maintenance) of adult-specific neurites was largely blocked, and many neurons subsequently disappeared altogether or ceased to express bursicon and *ccap-Gal4*. Because *foxo* overexpression under the *bursicon-Gal4* driver and all other IIS manipulations with the *ccap-Gal4* driver (below) did not result in substantial cell loss, we did not determine whether the loss in *ccap*>FOXO animals was due to possible neurotoxic functions of FOXO (Kanao et al., 2010; Siegrist et al., 2010).

### InR regulated metamorphic growth of the CCAP/bursicon neurons

**Fig. 2.**
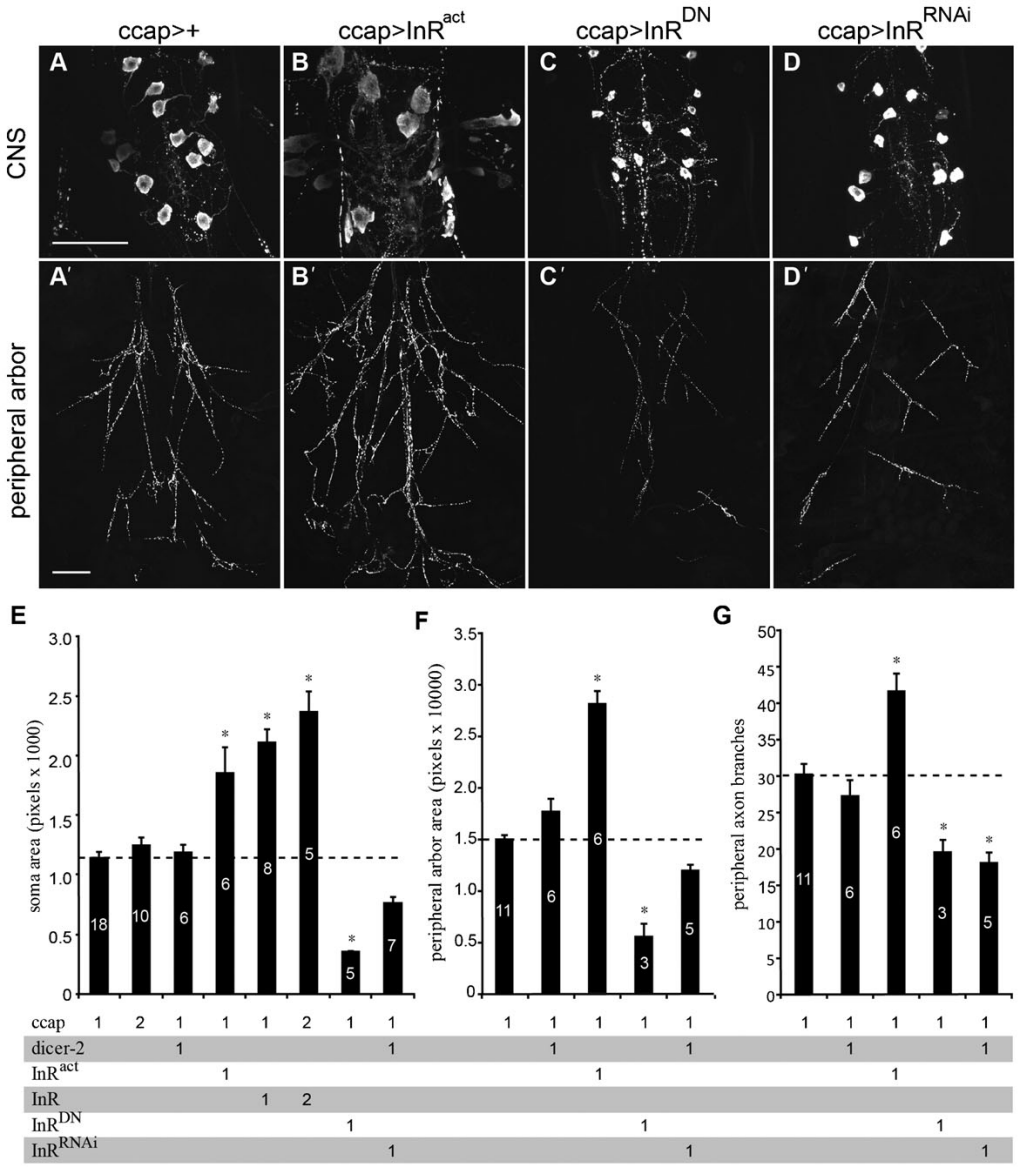
InR regulated metamorphic growth of CCAP/bursicon cell somata and peripheral axon arbor. (A–D) Cell-targeted expression of *InR^act^* in the CCAP/bursicon neurons increased soma size (B) and extent of the peripheral axon arbor (B′) (anti-bursicon immunostaining, stage P14 pharate adults). In contrast, expression of *InR^DN^* and *InR^RNAi^* produced smaller somata (C,D) and reduced the peripheral arbor (C′,D′). (E–G) The CCAP/bursicon somata size (E), area covered by the peripheral axon arbor (F), and number of axonal branches (G) were dependent on InR activity. One or more copies of each transgene were present in each genotype as indicated below the histograms: CCAP  =  *ccap-Gal4*; dicer-2  =  *UAS-dicer-2*; InR^act^  =  *UAS-InR^act^*; InR^DN^  =  *UAS-InR^DN^*; InR^RNAi^  =  *UAS-InR^RNAi^*. One-way ANOVAs and Tukey–Kramer *post-hoc* tests were performed on soma size (*P*<0.001), peripheral arbor area (*P*<0.001), and peripheral arbor branch number (*P*<0.001). Scale bars: 100 µm (A–D), 200 µm (A′–D′).

The IIS pathway negatively regulates FOXO. Specifically, Akt, a key downstream component of the pathway, phosphorylates FOXO, blocking its nuclear translocation and thus its transcriptional regulatory functions (Puig et al., 2003). Therefore, we tested whether co-overexpression of *foxo* and other genes that positively regulate the IIS pathway (*InR* and *PI3K*) restored normal development and function of the CCAP/bursicon neurons. For cellular analysis, we focused on the abdominal CCAP/bursicon neurons, which contain all of the efferent cells. When either *InR* or *PI3K* was overexpressed with *foxo*, all adult progeny had fully expanded wings, and the morphology of the CCAP/bursicon neurons was normal (supplementary material Fig. S6, and data not shown). These results confirmed that ectopic expression of InR and PI3K inhibited FOXO in the CCAP/bursicon neurons. However, these co-overexpression experiments did not address whether native IIS directly regulated this remodeling process. To address that question, we changed IIS levels through cell-targeted downregulation and upregulation of InR function and examined the effects of altered IIS on the CCAP/bursicon neurons at the pharate adult stage (Fig. 2). Downregulation of InR by expression of a dominant negative mutant of *InR* (*InR^K1409A^*, hereafter referred to as *InR^DN^*) (Wu et al., 2005; Tweedie et al., 2009) in the CCAP/bursicon neurons reduced the soma area to 30–52% of normal (Fig. 2C,E; supplementary material Fig. S4) and the peripheral axon arbor area to 38% of normal (Fig. 2C′,F). The number of peripheral axon branches was also reduced to 60% of normal (Fig. 2C′,G). RNA interference to *InR* (*InR^RNAi^*) (Shim et al., 2012) produced a similar reduction in the peripheral axon branch number (Fig. 2D′,G), but any effects on soma area and peripheral axon arbor were more modest (Fig. 2D,D′,E,F). Overexpression of *InR* or expression of a constitutively active mutant of *InR* (*InR^R418P^*, hereafter referred to as *InR^act^*) (Wu et al., 2005; Tweedie et al., 2009) led to a 208% increase in soma area (Fig. 2B,E). In addition, the area covered by the peripheral axon arbor was increased to 189% of controls (Fig. 2B′,F), and the number of branches in the axon arbor was increased to 140% of normal (Fig. 2B′,G). These results showed that CCAP/bursicon neuron soma and axon growth and axon branching during metamorphosis are strongly dependent on InR activity.

**Fig. 3.**
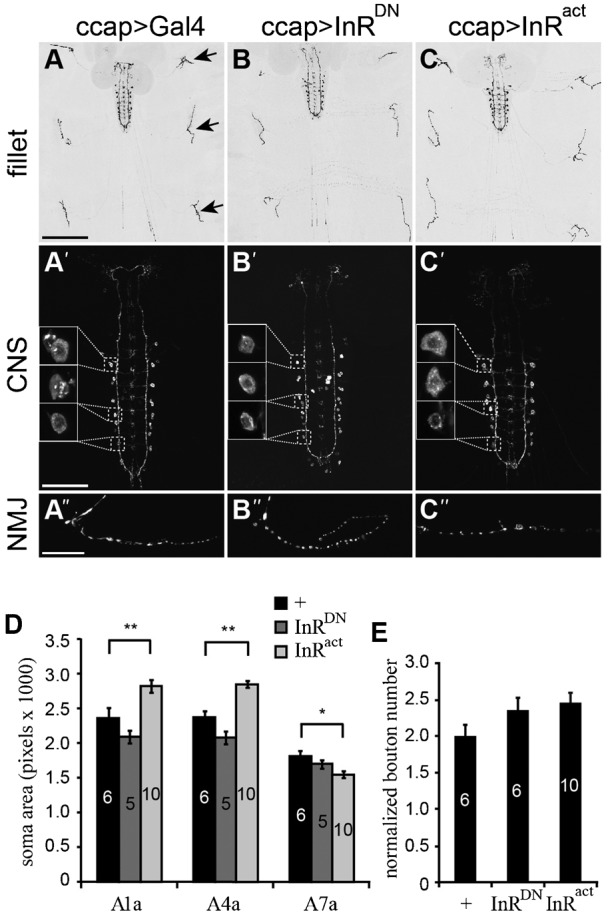
Larval growth of the CCAP/bursicon neurons was insensitive to loss of InR. (A–C) Cell-targeted expression of *InR^DN^* and *InR^act^* in the CCAP/bursicon neurons had little effect on the larval peripheral arbor (B,C), central projection pattern (B′,C′), or neuromuscular junctions (NMJ) (B″,C″) (anti-bursicon immunostaining on w3^rd^ larvae). The arrows in panel A show selected NMJs, and the insets in panels A′, B′ and C′ show the A1a, A4a, and A7a somata (from anterior to posterior). (D,E) Quantification of CCAP/bursicon cell soma size for A1a, A4a, and A7a (D) and normalized bouton number in abdominal segment 4 (E) for the genotypes in panels A–C. InR^act^ significantly affected the soma sizes of A1a, A4a, and A7a. In contrast, InR^DN^ had no effect on soma size (*P*>0.05). Both InR^DN^ and InR^act^ had no effect on bouton number (E). One-way ANOVAs and Tukey–Kramer *post-hoc* tests were performed on soma size of A1a (*P*<0.0001), A4a (*P*<0.0001), and A7a (*P*<0.05), and normalized bouton number (*P*>0.05,). Scale bars: 200 µm (A–C), 100 µm (A′–C′), 20 µm (A″–C″).

Since the major impacts of *foxo* overexpression in the CCAP/bursicon neurons were observed during metamorphosis (and not in larvae), we wondered whether altered InR function would also affect growth of these neurons in a stage-dependent manner. To test this hypothesis, we conducted anti-bursicon immunostaining on wandering 3^rd^ instar stage larvae expressing *InR^DN^* or *InR^act^* in the CCAP/bursicon neurons. The gross morphology of the larval CCAP/bursicon neurons was essentially unchanged following *InR^DN^* or *InR^act^* expression (Fig. 3).

We also examined the effects of InR manipulations on larval CCAP/bursicon neuron somata and NMJs. In the abdominal ganglia, there are eight pairs of abdominal CCAP/bursicon neurons on each side of the CNS. Within each ‘a/b’ neuron pair, the ‘a’ neuron has a higher level of bursicon expression than ‘b’ (Zhao et al., 2008). We measured the soma size of A1a (the ‘a’ cell in abdominal ganglion 1), A4a, and A7a, and NMJ bouton number in segment 4. Interestingly, InR^DN^ did not alter soma size (Fig. 3B′,D) or larval NMJ bouton number (Fig. 3B″,E). We obtained similar results with cell-targeted expression of *InR^RNAi^* with *Dcr-2* in the CCAP/bursicon neurons (supplementary material Fig. S7). These results indicate that IIS plays a minor role in soma and synapse growth of the CCAP/bursicon neurons during larval development. However, we did observe a significant increase in soma size with *InR^act^* expression in the cells in more anterior segments (Fig. 3C′,D). Thus, IIS pathway components are present and functional to some degree in larval CCAP/bursicon neurons, even if signaling through the pathway is not normally active until metamorphosis.

**Fig. 4.**
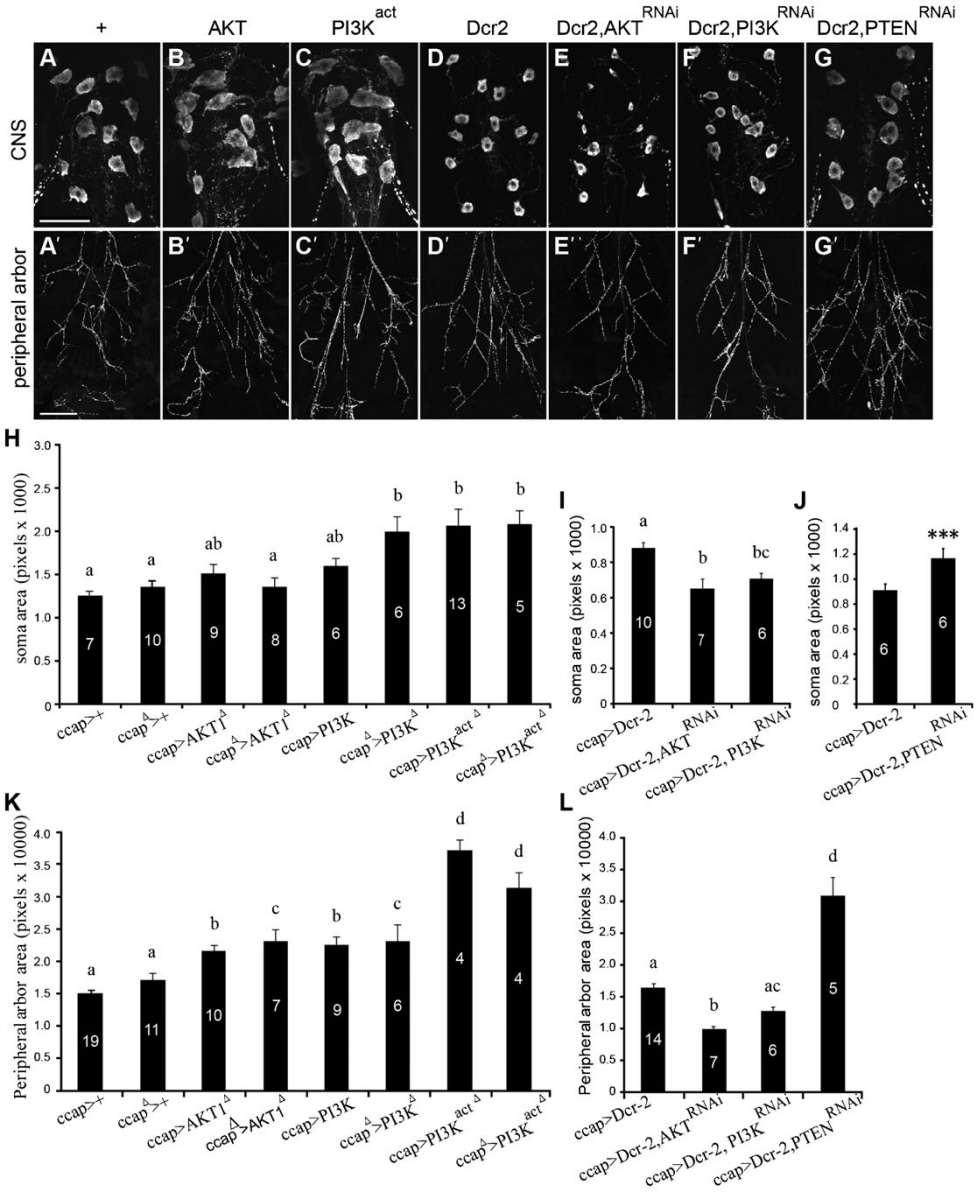
Akt, PI3K, and PTEN regulated metamorphic growth of CCAP/bursicon neurons. (A–G) Cell-targeted expression of *Akt*, *PI3K^act^*, and *PTEN^RNAi^* with *Dcr-2* in the CCAP/bursicon neurons increased soma size (B,C,G) and extent of the peripheral axon arbor (B′,C′,G′) (anti-bursicon immunostaining, stage P14 pharate adults). In contrast, expression of *Akt^RNAi^* and *PI3K^RNAi^* with *Dcr-2* produced smaller somata (E,F) and reduced the peripheral arbor (E′,F′). Controls had the *ccap-Gal4* driver alone (A) or *ccap-Gal4* with only *UAS-Dcr-2* (D). Each element used in the representative images in panels A–G was heterozygous. Scale bars: 50 µm (A–G), 200 µm (A′–G′). (H–L) The CCAP/bursicon soma size (H–J) and peripheral axon arbor area (K,L) were dependent on the activity of Akt, PI3K and PTEN. Each element used here was heterozygous unless marked with the symbol Δ, in which case the allele was homozygous. One-way ANOVAs and Tukey–Kramer *post-hoc* tests were performed on soma size (H) (*P*<0.0001), (I) (*P*<0.001), and (J) (*P*<0.001). Due to unequal variances, we used Kruskal–Wallis Multiple-Comparison Z-Value Tests with Bonferroni correction for statistical analysis of the peripheral arbor area in panels K (*P*<0.05) and L (*P*<0.05). Means labeled with different letters are significantly different (*P*<0.05).

We next tested whether additional components of the IIS pathway regulate the metamorphic growth of the CCAP/bursicon somata and peripheral axon arbor. First, we looked at the effects of PI3K, Akt, and Phosphatase and tensin homolog (PTEN), all of which are key upstream components of the IIS pathway (Wu and Brown, 2006). The kinases PI3K and Akt are positive regulators of the IIS pathway, whereas PTEN reduces IIS by inhibiting PI3K signaling. Increased IIS, through cell-targeted expression of PI3K, PI3K^act^, or PTEN^RNAi^, stimulated metamorphic growth of the cell bodies and peripheral axon arbor (Fig. 4). Increased levels of wild-type AKT (which may or may not lead to an increase in the amount of activated enzyme) produced a significant increase in peripheral arbor area, but we did not observe a significant increase in soma size. In contrast to the growth promoting effects of increases in IIS, decreases in IIS through RNAi to PI3K and Akt, suppressed neurite branching and growth of the CCAP/bursicon neuron somata (Fig. 4). These actions of PI3K, Akt, and PTEN in the CCAP/bursicon neurons further confirmed the role of insulin signaling in regulating the outgrowth of these neurons during metamorphosis.

### FOXO and TSC/TOR regulated metamorphic outgrowth of the CCAP/bursicon neurons

**Fig. 5.**
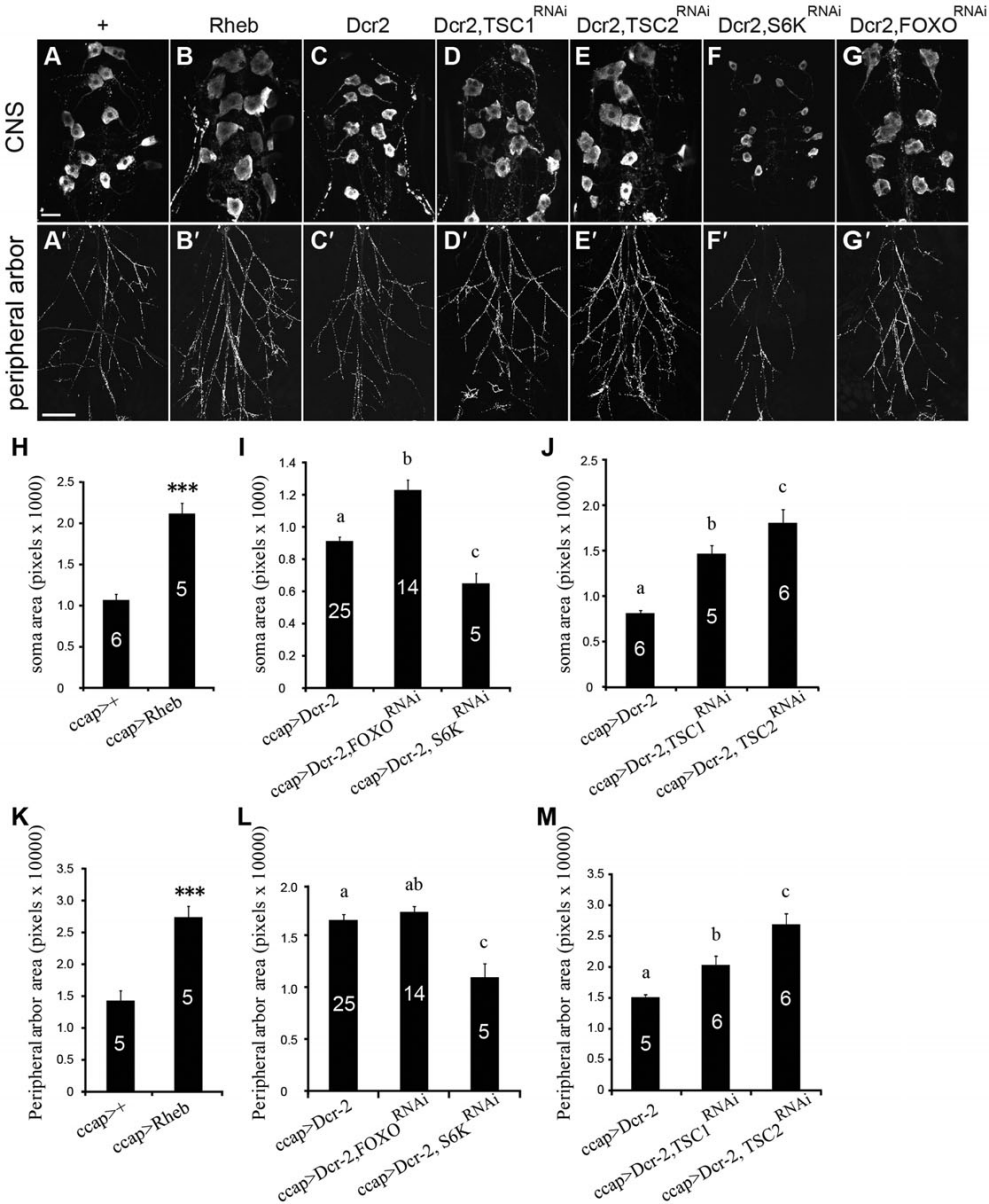
FOXO and Tor regulated metamorphic growth of CCAP/bursicon neurons. (A–E) Both *ccap-Gal4* driven expression of *Rheb* and RNAi for TSC1 and TSC2 (*TSC1^RNAi^* or *TSC2^RNAi^* with *Dcr-2*) increased the soma size (B,D,E) and the area covered by the peripheral axon arbor (B′,D′,E′). Panels A and C show *ccap-Gal4* driver-only controls and *UAS-Dcr-2, ccap-Gal4* controls, respectively. In contrast, expression of *S6K^RNAi^* with Dcr-2 decreased soma size (F) and the peripheral axon arbor (F′). Expression of *FOXO^RNAi^* with *Dcr-2* increased soma size (G), but not the peripheral axon arbor (G′). Cells were labeled by anti-bursicon immunostaining at P14 pharate adult stage. Each element used in the experiments was heterozygous. Scale bars: 20 µm (A–G), 200 µm (A′–G′). (H–M) Quantification of soma cross-sectional area (H–J) and peripheral axon arbor area (K–M) for CCAP/bursicon cells of the phenotypes shown in panels A–G. Student's *t*-tests were performed on the measurements shown in panels H (*P*<0.0001) and K (*P* = 0.0005). One-way ANOVAs with Tukey–Kramer *post-hoc* tests were performed on soma size measurements shown in panels I (*P*<0.0001) and J (*P*<0.001) and peripheral axon arbor measurements shown in panels L (*P*<0.001) and M (*P*<0.01). Means labeled with different letters are significantly different.

The IIS pathway regulates cellular growth through two prominent downstream targets of AKT, FOXO and Tuberous Sclerosis Complex (TSC)/Target of Rapamycin (TOR) (Jünger et al., 2003; Oldham and Hafen, 2003). Therefore, we asked whether FOXO and TSC/TOR both contributed to growth of CCAP/bursicon neurons during metamorphosis (Fig. 5). Given that *foxo* overexpression produced a strong phenotype (Fig. 1), we first examined the effects of *foxo* loss-of-function. Following CCAP/bursicon cell-targeted *foxo* RNAi, 98% of the adults displayed unexpanded wings (UEW), and the rest had partially expanded wings (PEW) (*n* = 54). In addition, soma size was increased (Fig. 5G,I), although the size of the peripheral axon arbor was unchanged (Fig. 5L). These results suggest that the FOXO arm of the IIS pathway is involved in the regulation of CCAP/bursicon soma growth during metamorphic remodeling, whereas other downstream IIS targets regulate outgrowth and branching of the CCAP/bursicon peripheral axon arbor. However, we cannot exclude the possibility that the RNAi to FOXO may have simply produced a weak FOXO loss-of-function phenotype.

We next examined whether TSC/TOR mediated the effects of IIS on axon growth and branching during metamorphosis. Activation of IIS inhibits TSC1/TSC2, a heterodimer that negatively regulates Rheb, an activator of the TOR complex (Saucedo et al., 2003; Wullschleger et al., 2006). TOR promotes growth through either phosphorylation of ribosomal protein kinase p-70-S6 (S6K) to increase protein synthesis, or inhibition of 4EBP to enhance translation (Jaeschke et al., 2002; Garami et al., 2003; Stocker et al., 2003). Activation of TOR through CCAP/bursicon-targeted expression of *UAS-Rheb* completely blocked adult wing expansion (*n* = 100), and it increased soma size and expanded the peripheral axon arbor in pharate adults (Fig. 5B,B′). Similarly, RNAi to TSC1 resulted in flies with 36% UEW and 64% expanded wings (*n* = 14) (the wing expansion phenotype for RNAi to TSC2 was not tested). We also observed a significant increase in soma size and peripheral axon arbor following RNAi to TSC1 and TSC2 (Fig. 5D,D′,E,E′,J,M).

Conversely, manipulations that reduced IIS led to decreased CCAP/bursicon neuron cell growth, although they also disrupted wing expansion. RNAi to S6K produced flies with 34% UEW, 11% PEW, and 55% expanded wings (*n* = 44), with a decrease in soma size and axon arborization (Fig. 5F,F′,I,L). These results reveal an important role of the TSC/TOR arm of the IIS pathway in stimulating metamorphic soma growth and axon outgrowth of the CCAP/bursicon neurons.

### IIS regulated growth of the Tv neurons during metamorphosis

We next asked whether IIS plays a broader role in regulating the metamorphic remodeling of other cell types. To address this question, we first studied another class of neuroendocrine cells, the Tv neurons, for which there are excellent cell markers (e.g. anti-RFamide antibodies) and in which neuronal remodeling during metamorphosis has been well characterized (Brown et al., 2006). We targeted expression of *UAS-foxo*, *UAS-InR^act^*, and *UAS-InR^DN^* to the Tv neurons and conducted anti-RFamide immunostaining (Benveniste et al., 1998) on pharate adult animals. Similar to the effects in the CCAP/bursicon neurons (Fig. 2B), expression of *InR^act^* led to a 28% increase in Tv neuron soma size and a 36% increase in the area covered by the adult peripheral axon arbor (supplementary material Fig. S8). In contrast, cell-targeted expression of *InR^DN^* in the Tv neurons significantly reduced soma size to 81% of normal, although there was no significant difference in the area covered by the axon arbor (supplementary material Fig. S8). The latter result may have underreported the effects of IIS on Tv neuron axon branching and outgrowth, since the arbor has a highly variable and branched architecture that is largely constrained on the surface of the CNS. Nevertheless, it is clear that changes in IIS led to changes in soma growth and neurite outgrowth, although the effects were modest in comparison to the changes seen in the CCAP/bursicon neurons (Fig. 2).

### IIS regulated the organizational growth, but not maintenance growth, of many peptidergic neurons

**Fig. 6.**
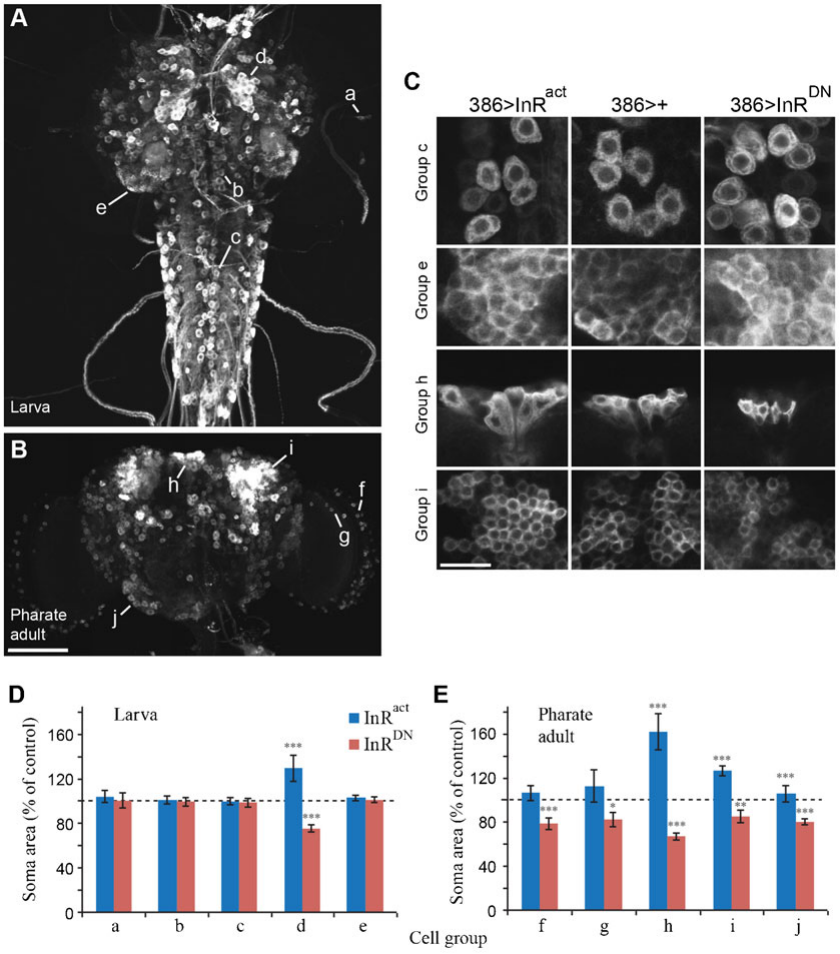
IIS affected the soma area of most neurons in pharate adults, but not in larvae. (A,B) Pan-peptidergic expression pattern of *386-Gal4,UAS-CD8::GFP* at the wandering 3^rd^ stage (A) and the pharate adult stage (B). We analyzed soma sizes (cross-sectional areas) for five larval groups of neurons labeled by a to e (A) and five pharate adult groups of neurons labeled by f to j (B). (C) Higher magnification views of selected neurons groups (c,e,h,i) expressing *InR^act^*, *InR^DN^*, or just the *386-Gal4* driver. Groups e and i are the mushroom body Kenyon cells. Groups d and h are the brain insulin-producing cells. (D,E) Soma sizes for the larval (D) and pharate adult (E) groups of neurons indicated in panels A and B following *InR^act^* or *InR^DN^* expression. Scale bars: 100 µm. One way ANOVA; Tukey–Kramer *post-hoc* tests were performed on soma size (*P<*0.0001) and peripheral axon arbor (*P*<0.0001). *n* = 5 for all three genotypes.

The effects of IIS on organizational growth of two groups of neurons (CCAP/bursicon and Tv), suggests that IIS may regulate the growth of many neurons during metamorphosis. To test this hypothesis, we manipulated InR activity under the control of *386-Gal4*, a pan-peptidergic cell driver (Taghert et al., 2001). It is difficult to separate and quantify changes in the neurites of single neurons within such a broad neuronal pattern, and the effects of IIS on soma size generally paralleled the ones on neurites in the CCAP/bursicon and Tv neurons (e.g. Fig. 2; supplementary material Fig. S8). Therefore, we measured soma size as a proxy for the effects of InR on growth of diverse peptidergic neurons in the *386-Gal4* pattern. Based on soma morphologies and locations, we selected five groups of neurons that were easily distinguished at the wandering 3^rd^ instar larval stage (groups a to e) and five groups of neurons that were identifiable at the pharate adult stage (groups f to j) (Fig. 6A,B). For example, the larval group c neurons had large, round somata located along the dorsal midline in the ventral nerve cord (Fig. 6A,C), while the pharate adult group h neurons, which are the insulin-producing cells (IPCs; data not shown), had large, triangular somata located in the medial protocerebrum of the brain (Fig. 6B,C). For each group, we measured the cross-sectional area of cells (visualized as two-dimensional confocal projections) labeled with the CD8::GFP marker. In larvae, four of five cell types displayed no change in soma size in response to InR^act^ or InR^DN^ (Fig. 6C,D). In contrast, all five groups of pharate adult neurons displayed marked changes in soma size (Fig. 6C,E). In general, the growth of most larval neurons appeared refractory to changes in IIS, whereas most neurons were highly responsive during metamorphosis. These results suggest that the stage-dependent regulation of CCAP/bursicon growth by IIS is representative of many different classes of peptidergic neurons.

### Putative local source of IIS for regulation of metamorphic growth of the CCAP/bursicon neurons

**Fig. 7.**
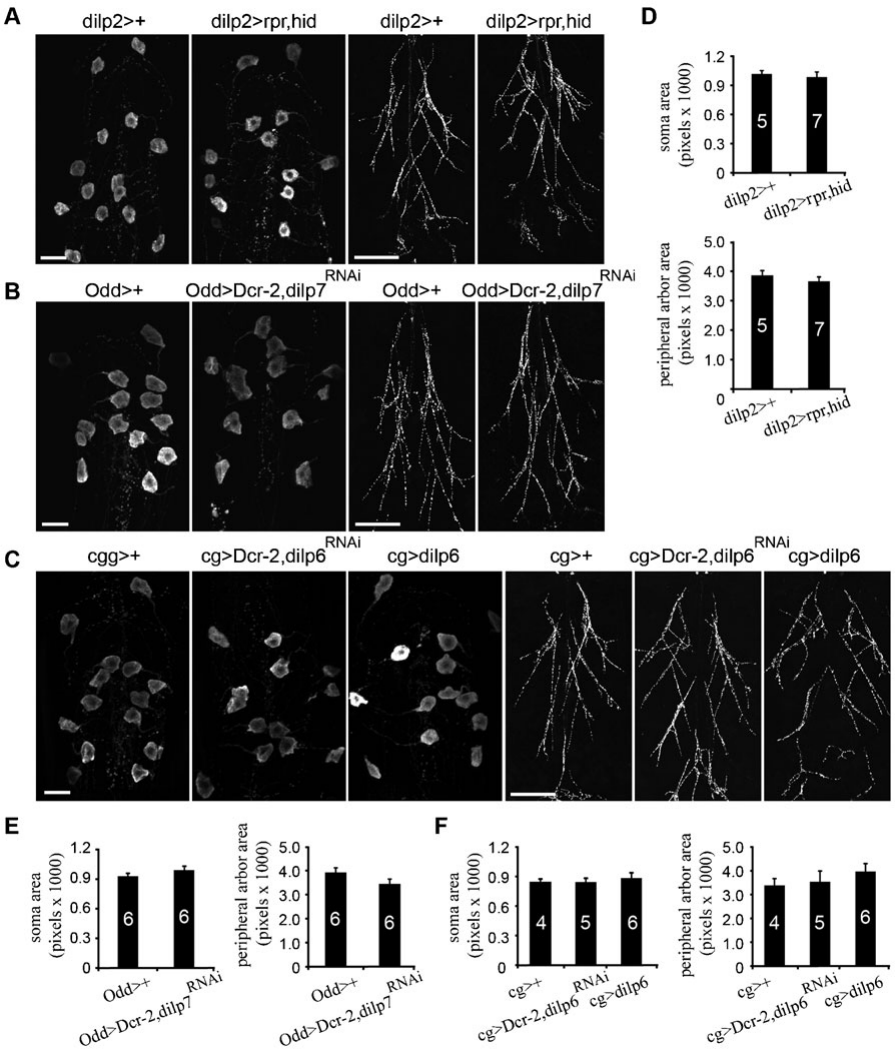
Elimination of circulating DILP sources did not affect metamorphic growth of the CCAP/bursicon neurons. (A–C) Confocal images of the CCAP/bursicon neuron somata and axon arbor following the ablation of three sources of circulating DILPs. Ablation of the brain IPCs was achieved through cell-targeted expression of *UAS-rpr, UAS-hid* driven by *dilp2-Gal4* (A). To downregulate DILP7, we targeted *dilp7^RNAi^* to the VNC dMP2 neurons with *Odd-Gal4* driver (B). Changes in the level of DILP6 in the fat body were achieved with the expression of *UAS-dilp6^RNAi^* or *UAS-dilp6* under the control of the fat body-specific driver, *cg-Gal4* (C). (D–F) Quantification of CCAP/bursicon cell soma size and peripheral axon arbor area for the genotypes in panels A–C. Student's *t*-tests and one-way ANOVA test were performed on soma size and peripheral axon arbor, respectively; none of the differences were statistically significant. Scale bars: 20 µm (CNS), 200 µm (peripheral arbor).

*Drosophila* insulin-like peptides are encoded by eight genes, *dilp1–8* (Brogiolo et al., 2001; Colombani et al., 2012; Garelli et al., 2012), which differ in their spatial and temporal expression patterns. To determine which DILP(s) regulate metamorphic growth of the CCAP/bursicon neurons, we tested three of the known or putative sources of circulating DILP hormones: the brain IPCs, the fat body, and the VNC IPCs (Fig. 7). The brain IPCs are seven pairs of cells in each brain hemisphere that synthesize DILP2, 3 and 5 and secrete these hormones into the hemolymph to regulate glucose homeostasis and growth (Brogiolo et al., 2001; Ikeya et al., 2002). We ablated the brain IPCs by expressing the cell death genes, *reaper (rpr)* and *hid* (Schetelig et al., 2011), under the control of the *dilp2* promoter (Rulifson et al., 2002). In this cross, only female progeny contained the *UAS-rpr* and *UAS-hid* transgenes. The male progeny were normal. In females, the developmental time from egg to adult was extended from 12 to 22 days at 25°C, and adult body size was substantially reduced (data not shown), consistent with earlier findings (Rulifson et al., 2002). Nevertheless, females displayed normal metamorphic growth of the CCAP/bursicon neurons (Fig. 7A,D), indicating that the brain IPCs were not necessary for this growth. We next tested DILP6, which is highly expressed in the fat body after the late 3^rd^ instar and secreted into the hemolymph to regulate growth during metamorphosis (Okamoto et al., 2009; Slaidina et al., 2009). We altered the level of DILP6 by expression of *UAS-dilp6* or *UAS-dilp6^RNAi^* with *UAS-Dcr-2* under the control of a fat body-specific driver, *cg-Gal4*. Neither of these *dilp6* manipulations had any effect on the CCAP/bursicon neurons (Fig. 7C,F), even though both of these genotypes alter post-feeding growth regulation (Slaidina et al., 2009). Finally, we examined the role of DILP7 in the putatively neuroendocrine dMP2 neurons, which are located in the posterior of the ventral nerve cord (Miguel-Aliaga et al., 2008; Yang et al., 2008). Targeted expression of *dilp7^RNAi^* with *Dcr-2* in the dMP2 neurons had no effect on the CCAP/bursicon somata or axon arbor (Fig. 7B,E), although the *dilp7^RNAi^* RNAi animals displayed a marked reduction in DILP7 peptide (supplementary material Fig. S9). Although we cannot exclude the possibility of compensatory DILP expression (see Discussion) or residual DILP signaling in the above genotypes, our results indicate that the metamorphic growth of the CCAP/bursicon neurons is not regulated by DILP2, 3, and 5 from the brain IPCs, DILP6 from the fat body, or DILP7 from the dMP2 neurons. These findings suggest that a local source of secreted insulin may regulate metamorphic CCAP/bursicon neuron growth.

## Discussion

### Stage-dependent effects of IIS on neuronal development

It is well established that IIS is crucial for regulating cell growth and division in response to nutritional conditions in *Drosophila* (Hietakangas and Cohen, 2009). However, most studies have focused on growth of the body or individual organs, and comparatively little is known about the roles of IIS during neuronal development, particularly in later developmental stages. *Drosophila* InR transcripts are ubiquitously expressed throughout embryogenesis, but are concentrated in the nervous system after mid-embryogenesis and remain at high levels there through the adult stage (Garofalo and Rosen, 1988). This suggests that IIS plays important roles in the post-embryonic nervous system. Recently, analysis of *Drosophila* motorneurons, mushroom body neurons, and IPCs has revealed important roles of PI3K and Rheb in synapse growth or axon branching (Knox et al., 2007; Howlett et al., 2008; Acebes et al., 2011; Zhao and Campos, 2012). These studies revealed some growth regulatory functions of IIS in the CNS, but they have not explored whether the control of neuronal growth by IIS is temporally regulated.

Here, we have shown that IIS strongly stimulates organizational growth of neurons during metamorphosis, whereas the effects of IIS on larval neurons are comparatively modest (Fig. 6). Recently, another group reported similar results in mushroom body neurons, in which the TOR pathway strongly promoted axon outgrowth of γ-neurons after metamorphic pruning (Yaniv et al., 2012). Expression of FOXO or reduction of InR function had no significant effect on larval growth of the CCAP/bursicon neurons (Figs 1, 3; supplementary material Fig. S7), or on the soma size of many other larval neurons (Fig. 6). Thus, while IIS has been shown to regulate motorneuron synapse expansion in larvae (Knox et al., 2007; Howlett et al., 2008), our findings indicate that IIS may not play a major role in regulating structural growth in many larval neurons. This is consistent with a recent report that concluded that the *Drosophila* larval CNS is insensitive to changes in IIS (Cheng et al., 2011).

When we used InR^act^ to activate IIS without ligand, we saw a modest but significant increase in the soma size of the more anterior CCAP/bursicon neurons during larval development (Fig. 3D). This result indicates that the IIS pathway is present and functional in these larval neurons, but the ligand for InR is either absent or inactive. During metamorphosis, unlike in larvae, downregulation of IIS by altering the level of InR or downstream components of the pathway significantly reduced CCAP/bursicon neuron growth (Figs 2, 4, 5). Thus, our results suggest that IIS is strongly upregulated during metamorphosis to support post-embryonic, organizational growth of diverse peptidergic neurons, and this activation may at least in part be due to the presence of as yet unidentified InR ligands during metamorphosis.

We attempted to identify this proposed InR ligand source by eliminating, in turn, most of the known sources of systemic DILPs (Fig. 7). None of these manipulations had any effect on metamorphic growth of the CCAP/bursicon neurons. These results are consistent with three possible mechanisms. First, there may be a compensatory IIS response to loss of some *dilp* genes. For example, a compensatory increase in fat body DILP expression has been observed in response to ablation of brain *dilp* genes (Grönke et al., 2010). Second, the growth may be regulated by another systemic hormone (e.g. DILP8) that was not tested, or by residual DILP peptides in the RNAi knockdown animals. Third, a local insulin source may be responsible for stimulating metamorphic outgrowth of the CCAP/bursicon neurons. Consistent with this view, a recent report showed that DILPs secreted from glial cells were sufficient to reactivate neuroblasts during nutrient restriction without affecting body growth, while overexpression of seven *dilp* genes (dilp1–7) in the IPCs had no effect on neuroblast reactivation under the same conditions (Sousa-Nunes et al., 2011). It seems likely that glia or other local DILP sources play an important role in regulating metamorphic neuron growth, but further experiments will be needed to test this model.

### Wing expansion defects after changes in IIS in the bursicon neurons

When we manipulated IIS in the CCAP/bursicon neurons, we observed changes in cell body size (and sometimes shape) and in the extent of branching in the peripheral axon arbor (Fig. 2). Although we focused our analysis of neurite growth on the peripheral axons, which are easily resolved in fillet preparations, we also observed corresponding changes in the size and complexity of the central CCAP/bursicon neuron arbor (data not shown). These IIS manipulations (both upregulation or downregulation) resulted in the above structural changes as well as wing expansion defects, suggesting that the normal connectivity of the CCAP/bursicon neurons was required for proper functioning of this cellular network. This model is consistent with the observation of two subsets of morphologically distinct bursicon-expressing neurons (the B_SEG_ and B_AG_ neurons), which are activated sequentially to control central and peripheral aspects of wing expansion (Peabody et al., 2008). The B_SEG_ neurons project widely within the CNS to trigger wing expansion behavior as well as secretion of bursicon by the B_AG_ neurons (Peabody et al., 2008). In turn, the B_AG_ neurons send axons into the periphery to secrete bursicon into the hemolymph to control the process of tanning in the external cuticle. Therefore, manipulation of IIS within these neurons, and the changes in morphology that result, may disrupt the wiring and function of this network. However, because we cannot rule out the possibility that these IIS manipulations also altered neuronal excitability, synaptic transmission, or neuropeptide secretion (Zhao et al., 2008), we relied on measurements of cellular properties (and not wing expansion rates) when assessing the relative effects of different IIS manipulations on cell growth.

### The roles of IIS on age- and context-dependent neuronal regenerative ability

Our results indicate that IIS is critical for organizational growth, which occurs during insect metamorphosis but is also seen during neuronal regeneration in other systems. However, the regenerative ability of many neurons is age-dependent and context-dependent (Selzer, 2003; Park et al., 2010); immature neurons possess a more robust regenerative capacity, while the regenerative potential of many mature neurons is largely reduced. In particular, the adult vertebrate CNS displays very limited regeneration, in marked contrast to the regeneration abilities displayed by the peripheral nervous system (Ferguson and Son, 2011). Recent studies in mice suggest that age-dependent inactivation of mTOR contributes to the reduced regenerative capacity of adult corticospinal neurons, and activation of mTOR activity through PTEN deletion promoted robust growth of corticospinal tract axons in injured adult mice (Liu et al., 2010). Our genetic experiments demonstrate a requirement for activity of TOR, as well as several other IIS pathway components both upstream and downstream of TOR, in controlling organizational growth of many peptidergic neurons. This suggests that under certain conditions, the activation of IIS may be a crucial component of the conversion of mature neurons to a more embryonic-like state, in which reorganizational growth either after injury or as a function of developmental stage is possible. Given the strong evolutionary conservation of these systems and the powerful genetic tools available to identify novel regulatory interactions in *Drosophila*, studies on the control of organizational growth in this species hold great promise for revealing factors that are crucial for CNS regeneration.

## Supplementary Material

Supplementary Material
