# Supplementary material for: Insulin signaling regulates neurite growth during metamorphic neuronal remodeling
Source: Biol Open. 2013 Dec 11;3(1):81–93. doi: 10.1242/bio.20136437 (PMC3892163; doi:10.1242/bio.20136437)
Supplement: Supplementary Material [file supp_3_1_81__index.html]

Insulin signaling regulates neurite growth during metamorphic neuronal remodeling — Insulin signaling regulates neurite growth during metamorphic neuronal remodeling — Supplementary Material 

# Insulin signaling regulates neurite growth during metamorphic neuronal remodeling

## bio.20136437 Supplementary Material

**Files in this Data Supplement:**

- Supplementary Material - Tingting Gu et al. doi: 10.1242/bio.20136437
