# Supplementary material for: Insulin signaling regulates neurite growth during metamorphic neuronal remodeling
Source: Biol Open. 2013 Dec 11;3(1):81–93. doi: 10.1242/bio.20136437 (PMC3892163; doi:10.1242/bio.20136437)
Supplement: Supplementary Material [file supp_bio.20136437_bio.20136437-s1.pdf]

Supplementary Material

Tingting Gu et al. doi: 10.1242/bio.20136437

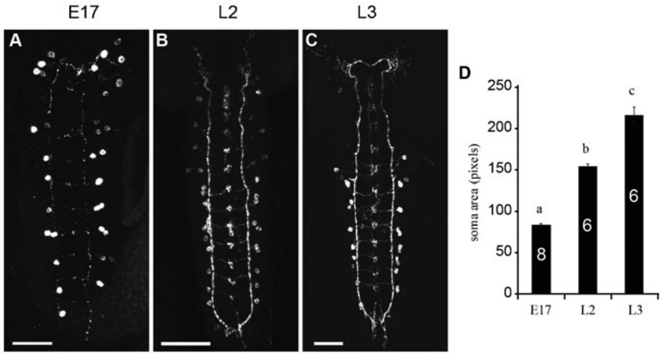

Fig. S1. The CCAP/bursicon cells grow but maintain a largely constant gross morphology during larval development. (A–C) CCAP/bursicon neurons from embryonic stage 17 (A), the second larval instar (B), and the wandering 3rd larval instar (C). (D) Quantification of soma size for CCAP/bursicon cells of the genotypes shown in panels A–C. From embryonic stage 17 to the wandering 3rd instar larval stage, the soma size (cross-sectional area) of the CCAP/bursicon neurons was increased over 2.5-fold. Bars labeled with different letters are significantly different ($P<0.0001$, one way ANOVA; Tukey–Kramer *post-hoc* test). Scale bars: 20 µm (A), 50 µm (B), 100 µm (C).

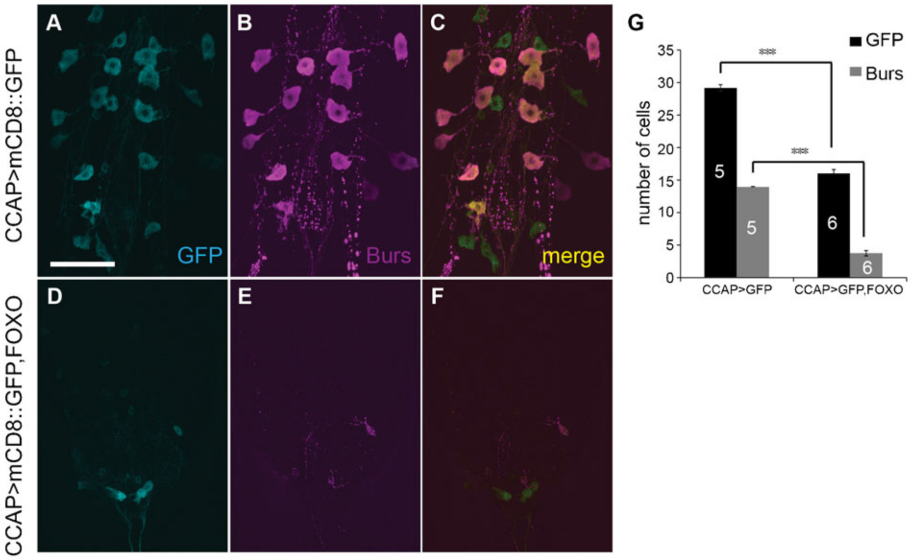

Fig. S2. Overexpression of *foxo* caused loss of CCAP- and bursicon-expressing neurons at the pharate adult stage. (A–F) *CCAP-Gal4*-driven co-expression of *UAS-mCD8::GFP* and *UAS-foxo* led to loss of neurons in stage P14 pharate adults, coupled with a significant reduction in soma and central neurite growth in the remaining neurons (D–F) (cyan, anti-GFP immunostaining; magenta, anti-bursicon immunostaining). Most remaining neurons expressed GFP but not bursicon. The control genotype (A–C) was *CCAP-Gal4, UAS-mCD8::GFP*. Scale bar: 50 µm. (G) Counts of CCAP neuron somata (GFP, black bars) and bursicon neuron somata (anti-bursicon, gray bars) for the genotypes in panels A–F ($P<0.0001$, Student's *t*-tests).

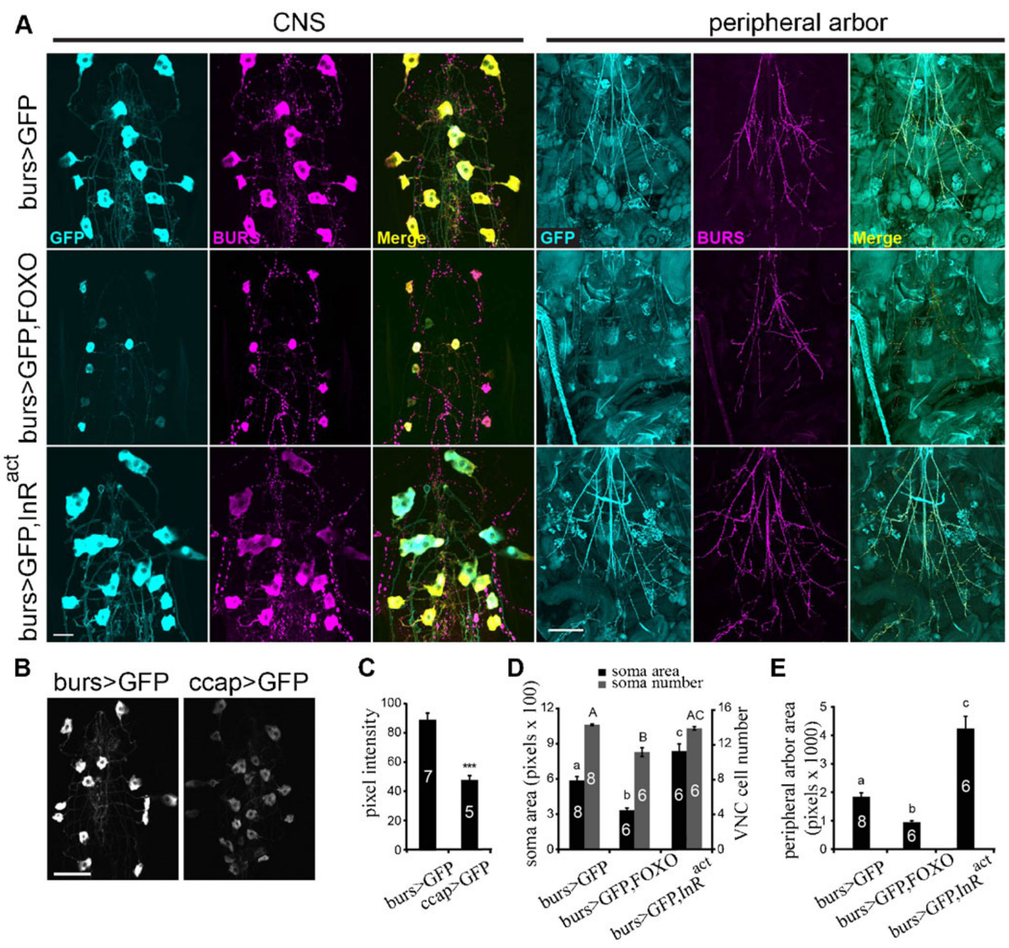

Fig. S3. Expression of *foxo* or *InR*^{act} driven by *burs-Gal4* altered metamorphic growth of the CCAP/bursicon cells. (A) *bursicon-Gal4*-driven co-expression of *UAS-mCD8::GFP* and *UAS-foxo* caused a significant reduction in soma and peripheral arbor growth and the number of visible somata (middle panels) at the pharate adult stage. In contrast, expression of *InR*^{act} significantly increased soma size and the area covered by the peripheral axon arbor (lower panels). There was substantial overlap of the CD8::GFP (cyan) and anti-bursicon immunostaining (magenta) signals, and strong CD8::GFP expression persisted in the somata, suggesting that the reduction of peripheral axon arbor area with *foxo* overexpression was not due to the loss of cell markers. (B) The expression level of CD8::GFP driven by *bursicon-Gal4* was higher than the one driven by *ccap-Gal4*. (C–E) Quantification of GFP fluorescence intensity (C), soma area (D, black bars), soma number (D, gray bars), and peripheral arbor area (E) of the CCAP/bursicon cells shown in genotypes in panels A and B. Means labeled with different letters were significantly different. A Student's *t*-test was performed on GFP intensity ($P<0.001$). One-way ANOVAs and Tukey–Kramer *post-hoc* tests were performed on soma size ($P<0.001$), soma number ($P<0.001$), and peripheral arbor area ($P<0.001$). Scale bars: 20 µm (A, CNS), 200 µm (A, peripheral arbor), 50 µm (B).

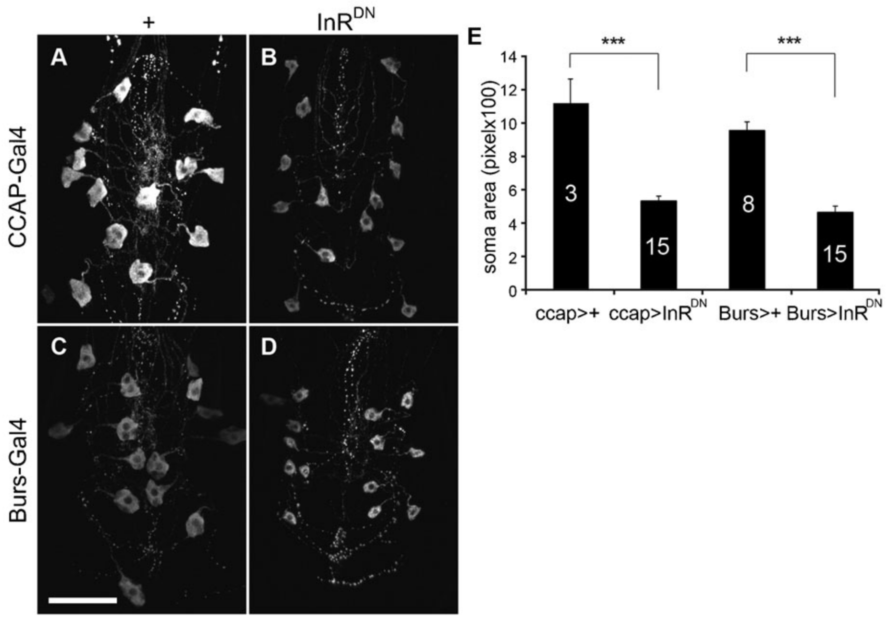

Fig. S4. *CCAP-Gal4* and *Burs-Gal4* displayed equivalent efficacy when driving *InR*^{DN} in the CCAP/bursicon neurons. (A–D) Expression *UAS-InR*^{DN} driven by *CCAP-Gal4* (A,B) or *Burs-Gal4* (C,D) significantly reduced soma size (B,D) in stage P14 pharate adults (anti-bursicon immunostaining). (A) *CCAP-Gal4* driver-only controls. (C) *Burs-Gal4* driver-only controls. Scale bar: 50 µm. (E) *InR*^{DN} expressed under the control of the *CCAP-Gal4* and *Burs-Gal4* drivers had similar effects on soma size of the CCAP/bursicon neurons ($P<0.0001$, Student's *t*-tests).

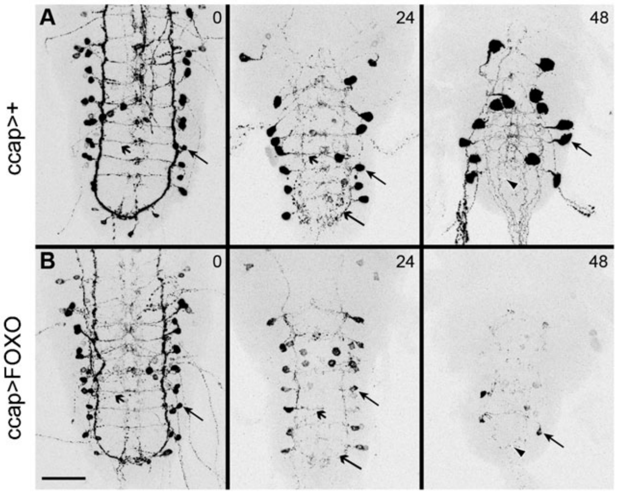

Fig. S5. Overexpression of *foxo* disrupted metamorphic outgrowth of the CCAP/bursicon cells. Anti-bursicon immunostaining of the ventral nerve cord at 0, 24 and 48 hours after puparium formation (APF). In contrast to the controls (A, with only the *ccap-Gal4* driver), CCAP/bursicon cell-targeted expression of *foxo* (B) disrupted soma growth (solid arrows) and outgrowth of adult-specific neurites (solid arrowheads). A similar extent of pruning of the larval neurites was observed at the 24 hour time point in the two genotypes. Feathered arrows, lateral longitudinal tracks; feathered arrowheads, midline arbor. Scale bar: 100 µm.

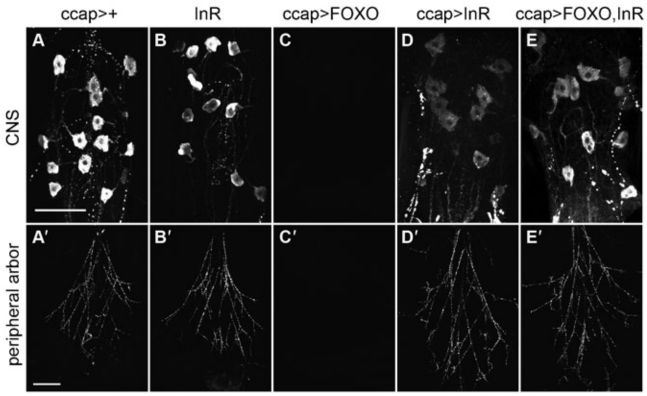

Fig. S6. InR inhibited the effects of FOXO in the CCAP/bursicon cells. Cell-targeted expression of InR in the CCAP/bursicon neurons completely rescued the cell loss and cell growth phenotypes induced by *foxo* overexpression. (A,A′) *ccap-Gal4* driver-only controls. (B,B′) *UAS-InR*-only controls. (C,C′) *ccap-Gal4; UAS-foxo*. (D,D′) *ccap-Gal4; UAS-InR*. (E,E′) *ccap-Gal4; UAS-InR, UAS-foxo*. Scale bars: 50 µm (A–E), 200 µm (A′–E′).

Biology Open

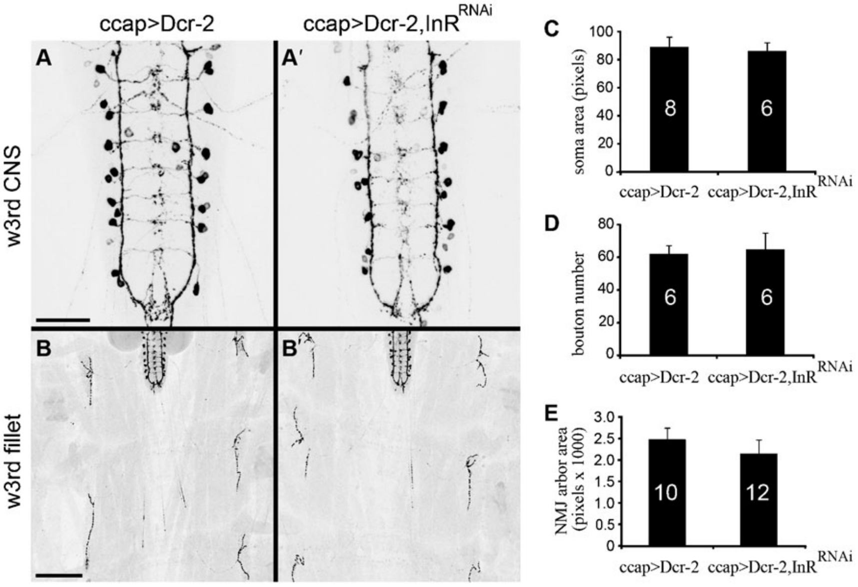

Fig. S7. InR^{RNAi} did not reduce soma or synapse growth in larval CCAP/bursicon cells. (A,B,A′,B′) Cell-targeted expression of *InR^{RNAi}* with *Dcr-2* in the CCAP/bursicon neurons had no effect on the general morphology of the CCAP/bursicon neuron somata and CNS projections (A,A′), peripheral axon projections (B,B′), or neuromuscular junction (NMJ)-like endings (anti-bursicon immunostaining on w3rd larvae). (A,B) *UAS-Dcr-2*, *CCAP-Gal4* controls. (A′,B′) *UAS-Dcr-2*, *CCAP-Gal4/UAS-InR^{RNAi}*. Scale bars: 100 µm (A,A′), 200 µm (B,B′). (C–E) InR^{RNAi} had no significant effect on soma area (C) ($P>0.05$, Student's *t*-test), bouton number (D) ($P>0.05$, Student's *t*-test), or NMJ bouton area (E) ($P>0.05$, Student's *t*-test).

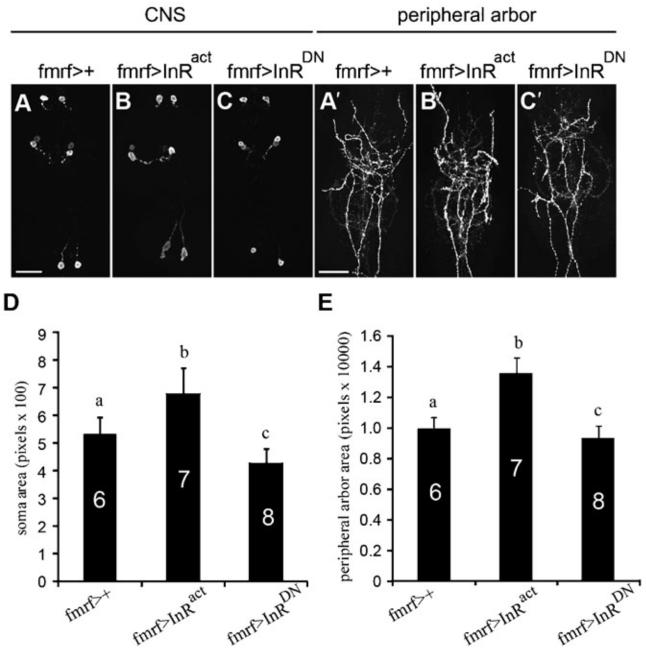

Fig. S8. Moderate effects of IIS on the metamorphic growth of Tv neurons. (A–C) Cell-targeted expression of *InR^{act}* or *InR^{DN}* in the Tv neurons changed the soma size (B,C) and the area covered by the peripheral axon arbor (B′,C′) (anti-bursicon immunostaining, stage P14 pharate adults). (A,A′) *fmrf-Gal4* driver-only controls. Scale bars: 100 µm. (D,E) Altered InR activity significantly influenced the soma size of Tv neurons (D) and the peripheral axonal arbor area (E). One-way ANOVA; Tukey–Kramer *post-hoc* tests were performed on soma size ($P<0.0001$) and peripheral axon arbor ($P<0.01$). Means labeled with different letters are significantly different.

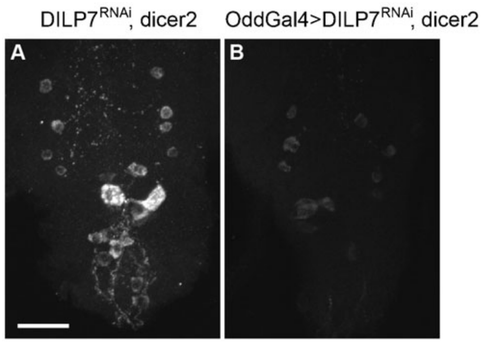

Fig. S9. Reduced levels of DILP7 peptide in the dMP2 neurons of *Odd>dilp7^{RNAi}*, *dicer-2* animals. (A,B) Stage P14 pharate adults expressing *UAS-dilp7^{RNAi}* with *UAS-dicer2* under the control of *Odd-Gal4* displayed reduced levels of DILP7 peptide (B) compared to *UAS-dilp7^{RNAi}*, *UAS-dicer2*-only controls (A) (anti-DILP7 immunostaining, stage P14 pharate adults). Scale bar: 25 µm.
